# Severe Coronary Artery Disease Disguised as Myocarditis

**DOI:** 10.7759/cureus.4159

**Published:** 2019-02-28

**Authors:** Varun Tandon, Manish Kumar, Christian M Mosebach, Aysha A Tandon

**Affiliations:** 1 Internal Medicine, University of Connecticut, Farmington, USA

**Keywords:** coronary artery disease, viral myocarditis, nstemi

## Abstract

Serum troponin is a marker of cardiac myocyte damage that is typically used to assess for myocardial infarction in the setting of acute coronary syndrome. However, many conditions, including cardiomyopathy, pulmonary embolism, or myocarditis, can cause an elevation in serum troponin. The most common use of this tool is to determine whether acute coronary syndrome (ACS) is occurring, but other differentials include cardiomyopathy, pulmonary embolism, and even acute heart failure. We present the case of a patient who presented with symptoms consistent with viral myocarditis but ultimately was found to have severe coronary artery disease (CAD). A 33-year-old Caucasian male with no cardiac risk factors other than a five-pack year smoking history, presented with progressively worsening upper respiratory symptoms, including sore throat and a non-productive cough that began a few weeks ago. These symptoms were associated with fevers, and 24 hours prior to admission, he developed intermittent chest pain at rest, radiating to the back, worsening in the supine position. In the emergency room (ER), the patient was found to have an elevated serum troponin of 15.61 ng/L (normal <0.05 ng/L). The electrocardiogram (EKG) showed T-wave inversions in the lateral leads. Based on his presentation and age, there was a high suspicion of viral myocarditis. However, non-ST elevation myocardial infarction (NSTEMI) had not yet been ruled out and the patient was started on started on a heparin infusion per the ACS protocol. A transthoracic echocardiogram showed wall motion abnormalities with low-normal left ventricular ejection fraction. A coronary angiogram showed severe CAD and he underwent staged a percutaneous coronary intervention with the resolution of symptoms. CAD and viral myocarditis, at times, can share common presenting symptoms, EKG changes, and laboratory findings. Out of all possible diagnoses, an elevation in serum troponin correlates to an MI up to 60% of the time. Myocarditis is the second leading cause of troponin elevation and accounts for 25% of cases. We highlight this case to discuss the importance of maintaining a broad differential and pursuing complete work-up when treating younger patients with chest pain and elevated serum troponin who lack typical risk factors for CAD.

## Introduction

Serum troponin is a marker of cardiac myocyte damage. It was originally developed and still is typically used to screen for myocardial infarction (MI) [1]. It was later noted that other conditions, such as cardiomyopathy, pulmonary embolism, and myocarditis, can also lead to elevated troponin levels [2]. It is important to understand the pathophysiology of troponin release from cardiac myocytes in order to help delineate the underlying cause. We present a case of a patient who presented with symptoms consistent with viral myocarditis but was found to have severe coronary artery disease (CAD).

## Case presentation

**Figure 1:**
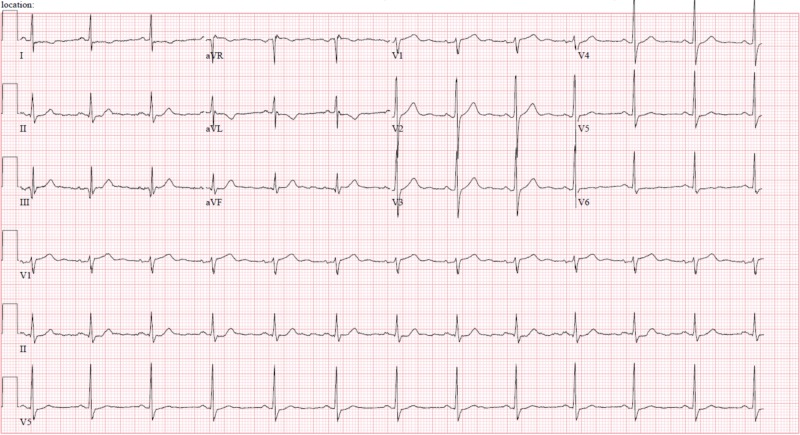
Electrocardiogram on presentation to the emergency room

**Figure 2:**
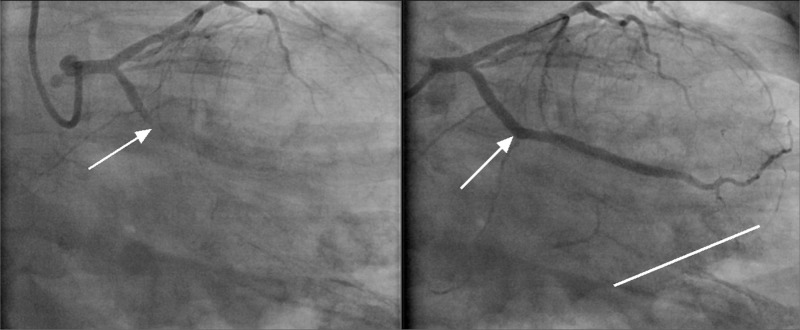
RAO caudal view of the left circumflex artery Left image: There is occlusion in the proximal segment of the left circumflex with a tapered occlusion site as indicated by an arrow; Right image:* *Drug-eluting stent placement with a return of flow through the artery, with an arrow demonstrating previous occlusion site. RAO: right anterior oblique

**Figure 3:**
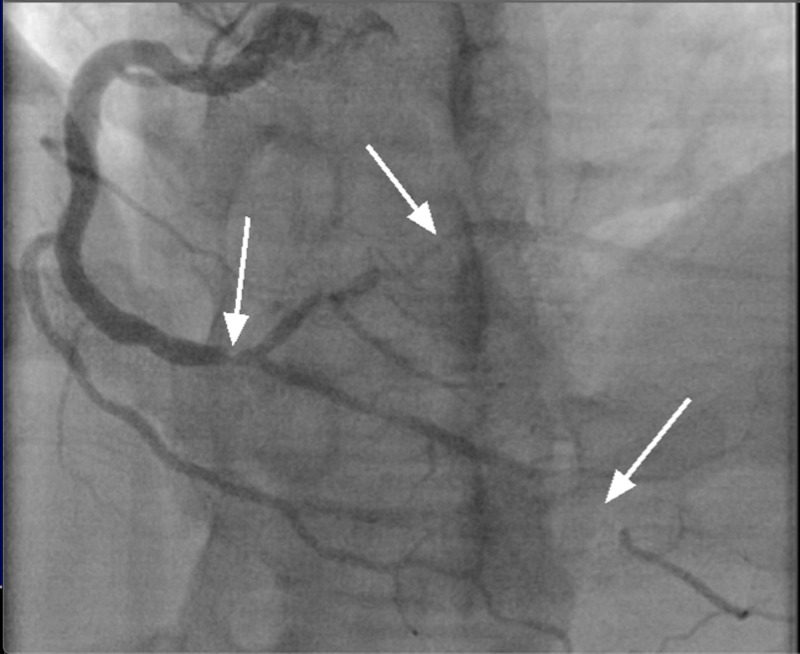
LAO of the right coronary artery Arrows pointing to critical disease in the right coronary artery with occlusion of the posterior descending artery and subtotal occlusion of the posterolateral artery LAO: left anterior oblique

A 33-year-old Caucasian male with no identifiable cardiac risk factors other than a five-pack-year smoking history, presented with fevers, body aches, upper respiratory symptoms, and chest pain. Upper respiratory symptoms, progressively worsening, began a few weeks prior to presentation with sore throat, rhinorrhea, lacrimation, and non-productive cough. Chest pain began 24 hours prior to presentation, which was intermittent and located in the left upper chest, radiating to the back and down his left arm. This pain was exacerbated by lying flat or getting up in a certain position but not with exertion. He denied any recent long-distance travel or driving. In the ER, the patient was noted to have low-grade fevers but was hemodynamically stable. The exam was only remarkable for mild left upper chest tenderness. Workup in the ER revealed leukocytosis of 15000 u/L (normal 3800-10600 u/L) and serum troponin of 15.61 ng/L (normal <0.05 ng/L). Electrocardiogram revealed T-wave inversions in the lateral leads (Figure 1). He was admitted with a provisional diagnosis of viral myocarditis. Although less likely, as NSTEMI was not ruled out, he was started on heparin drip per the acute coronary syndrome (ACS) protocol. A transthoracic echocardiogram showed inferior, inferolateral, and inferoseptal wall motion abnormality, with a low-normal left ventricular ejection fraction. The coronary angiogram demonstrated an occluded left circumflex artery and obtuse marginal (Figure 2) and critical disease of the right coronary artery with occlusion of the posterior-descending artery and subtotal occlusion of the posterolateral branch (Figure 3). It was decided to undergo percutaneous intervention (PCI) on the lesion within the left circumflex (Figure 2: right image). The patient was brought back later to the catheterization lab for staged PCI with a resolution of symptoms.

## Discussion

Viral myocarditis has a variety of presenting symptoms and, therefore, it can be difficult to differentiate this diagnosis from CAD. The clinical picture is further complicated by similar findings of elevated serum troponin in both cases. However, the underlying pathophysiology of viral myocarditis differs greatly from that of CAD. Viral myocarditis is a disease of the myocardium caused by common viral infections or post viral immune-mediated responses [3]. The virus gains entry via various receptors on myocytes and replicates, leading to myocyte necrosis and release of intracellular antigens into the surround extracellular space. This release causes the migration of the macrophages, natural killer (NK) cells, and T-lymphocytes. Over weeks to months, T-lymphocytes can continue to attack the myocardium due to viral molecular mimicry, leading to further damage and decreased contractile force. Most patients regain cardiac function once the viral infection has cleared, but chronic remodeling, causing permanent cardiomyopathy, can occur [3-5]. It is possible for patients to have both CAD and viral myocarditis, and the presence of CAD by itself does not exclude viral myocarditis [6]. It is hypothesized that this coexistence may occur due to the inflammatory process caused by viral myocarditis leading to the destabilization of atherosclerotic plaques [3,6-7].

Younger patients that present with chest pain and elevation in troponin should be evaluated for CAD due to the high morbidity and mortality associated with the delay in providing systemic anticoagulation and revascularization in the setting of an NSTEMI. Although younger patients may lack risk factors for CAD, an elevation in serum troponin correlates to myocardial infarction 60% of the time [2]. Of the remaining non-MI troponin elevation, myocarditis is the leading cause 25% of the time [2,8]. Additional differentials must also be considered. In previously healthy and young patients, differentials of pulmonary embolism, infiltrative disorders, cardiomyopathy, and even strenuous exercise must be included. History and physical exam must also be inclusive to the pretest probability and the likelihood for the need for the further exploration of these differentials. In patients who have a history of drug abuse, vasospastic angina may be a possibility but aortic dissection should also be a consideration.

If viral myocarditis is strongly suspected, cardiovascular magnetic resonance (CMR) imaging is helpful in confirming the diagnosis. Viral polymerase chain reaction (PCR) from the serum may help determine the culprit virus. Although the gold standard for diagnosis is an endomyocardial biopsy, with high specificity, the sensitivity is low, and there are significant risks associated with biopsy. Due to these disadvantages, CMR is the preferred method unless the patient, for some reason, is unable to undergo CMR [9].

This case highlights the fact that it can be difficult, at times, to differentiate myocarditis and CAD upfront, especially in young patients with a lack of risk factors, and that a broad differential should be considered in elevated troponin in young patients. In the absence of timely systemic anticoagulation and revascularization, NSTEMI is associated with high mortality and morbidity [10]. Even if the presentation is consistent with viral myocarditis, systemic anticoagulation should be initiated, unless there is a strong reason not to, and an ischemic evaluation, preferably a coronary angiogram, should also be performed.

## Conclusions

Serum troponin is a marker of cardiac myocyte damage. Troponin is frequently used to screen for MI, with MI as the leading cause of elevated serum troponin. However many other diseases, including myocarditis, can mimic MI and present with an elevation in troponins, but this is also useful in determining other cardiac pathologies. Myocarditis may result in troponin elevation due to inflammation within the myocardium. It is also important to recognize that the clinical presentation of viral myocarditis is widely variable and may present similarly to that of acute coronary syndrome. When a young patient presents with concerns of myocarditis, it is important to still consider acute coronary syndrome in the differential. This case highlights the difficulty, at times, to differentiate myocarditis and CAD. Even if the presentation is consistent with viral myocarditis, systemic anticoagulation should be initiated unless there is a strong reason not to and an ischemic evaluation, preferably a coronary angiogram, should be performed.
